# Association of anti-RNA polymerase III autoantibodies and cancer in scleroderma

**DOI:** 10.1186/ar4486

**Published:** 2014-02-14

**Authors:** Pia Moinzadeh, Carmen Fonseca, Martin Hellmich, Ami A Shah, Cecilia Chighizola, Christopher P Denton, Voon H Ong

**Affiliations:** 1 Centre for Rheumatology and Connective Tissue Diseases, University College London (UCL) Medical School, Royal Free Hospital, Pond Street, London NW3 2QG, UK; 2 Department of Dermatology, University of Cologne, Kerpener Str. 62, 50924 Cologne, Germany; 3 Institute of Medical Statistics, Informatics and Epidemiology, University of Cologne, Kerpener Str. 62, 50924 Cologne, Germany; 4 Division of Rheumatology, Johns Hopkins University School of Medicine, Johns Hopkins Scleroderma Center, 5501 Hopkins Bayview Circle, Room 1B.32, Baltimore, MD 21224, USA; 5 Rheumatology Division, Department of Clinical Sciences and Community Health, University of Milan, Istituto G. Pini Piazza C. Ferrari, 1 – 20122 Milan, Italy

## Abstract

**Introduction:**

We assessed the profile and frequency of malignancy subtypes in a large single-centre UK cohort for patients with scleroderma (systemic sclerosis; SSc). We evaluated the cancer risk among SSc patients with different antibody reactivities and explored the temporal association of cancer with the duration between SSc onset and cancer diagnosis.

**Methods:**

We conducted a retrospective study of a well-characterised cohort of SSc patients attending a large tertiary referral centre, with clinical data collected from our clinical database and by review of patient records. We evaluated development of all cancers in this cohort, and comparison was assessed with the SSc cohort without cancer. The effect of demographics and clinical details, including antibody reactivities, were explored to find associations relevant to the risk for development of cancer in SSc patients.

**Results:**

Among 2,177 patients with SSc, 7.1% had a history of cancer, 26% were positive for anticentromere antibodies (ACAs), 18.2% were positive for anti-Scl-70 antibodies and 26.6% were positive for anti-RNA polymerase III (anti-RNAP) antibody. The major malignancy cancer subtypes were breast (42.2%), haematological (12.3%), gastrointestinal (11.0%) and gynaecological (11.0%). The frequency of cancers among patients with RNAP (14.2%) was significantly increased compared with those with anti-Scl-70 antibodies (6.3%) and ACAs (6.8%) (*P* < 0.0001 and *P* < 0.001, respectively). Among the patients, who were diagnosed with cancer within 36 months of the clinical onset of SSc, there were more patients with RNAP (55.3%) than those with other autoantibody specificities (ACA = 23.5%, *P* < 0.008; and anti-Scl-70 antibodies = 13.6%, *P* < 0.002, respectively). Breast cancers were temporally associated with onset of SSc among patients with anti-RNAP, and SSc patients with anti-RNAP had a twofold increased hazard ratio for cancers compared to patients with ACAs (*P* < 0.0001).

**Conclusions:**

Our study independently confirms, in what is to the best of our knowledge the largest population examined to date, that there is an association with cancer among SSc patients with anti-RNAP antibodies in close temporal relationship to onset of SSc, which supports the paraneoplastic phenomenon in this subset of SSc cases. An index of cautious suspicion should be maintained in these cases, and investigations for underlying malignancy should be considered when clinically appropriate.

## Introduction

Systemic sclerosis (scleroderma, or SSc) is a heterogeneous, multisystem connective tissue disease that arises as a consequence of a complex interplay of altered immunologic processes involving vascular endothelial cell damage and excessive activation of fibroblasts, culminating in skin sclerosis and fibrotic changes of affected visceral organs.

The association between SSc and increased cancer risk has been reported in several case series, although this association has not been consistently demonstrated across all studies [1]. Researchers who have conducted recent meta-analyses have reported increased numbers of solid organ malignancies in SSc patients, including lung cancers but not breast cancers. Investigators in other studies, however, have suggested that lung cancers are considered to be common in patients with SSc [1-6]. The significant heterogeneity and variability in the cancer subtypes reported in these studies may be a consequence of limited patient numbers, patient characteristics and study design. Moreover, the actual role of SSc in the development of cancer remains unclear. For example, it is debatable if SSc patients with severe interstitial lung disease have an increased risk of developing lung cancer.

Few studies have examined the temporal relationship of cancers to the onset of SSc, and the current literature that links breast cancer to the onset of SSc remains contradictory [7,8]. The role of autoantibodies and malignancies in other autoimmune rheumatic diseases, however, has been well-described, such as in large population-based studies for inflammatory myositis. Anti-MJ/NXP2 and anti-p155/140 antibodies have been described in cancer-associated myositis. With regard to the latter, its high negative predictive value is potentially helpful to rule out the presence of occult malignancy in patients with dermatomyositis [9,10]. In contrast, only a few small case series have indicated that the presence of hallmark SSc-specific antibodies, anticentromere antibodies (ACAs) and anti-Scl-70 antibodies, correlates with cancer in SSc, but this finding has not been consistently replicated in other studies. Shah *et al*. recently reported that anti-RNA polymerase III (anti-RNAP) antibodies may be associated with malignancy in a small cohort of five patients with early-stage SSc [8]. Airò *et al*. also described similar clustering of cancer associated with the onset of SSc in a small sample of patients [7]. However, this observation has yet to be confirmed independently in a large cohort of patients. A better understanding of this relationship between antibody specificity and cancer in SSc is necessary to optimise follow-up and surveillance of these patients.

We evaluated the frequency of cancer subgroups in a large, single-centre UK cohort of patients with SSc. The demographic and clinical characteristics of this cohort were compared with matched cases without a history of cancer. The risk of cancer was assessed across patients with different autoantibody status, and the temporal relationship between cancer diagnosis and clinical onset of SSc was explored.

## Methods

We conducted a retrospective study of patients attending our centre. All of our patients met the classification criteria of LeRoy as having limited cutaneous SSc (lcSSc) or diffuse cutaneous SSc (dcSSc) [11-13]. Approval for this study was obtained from the London Hampstead National Research Ethics Services Committee, and all individuals consented to use of their clinical and laboratory data for the purposes of research.

To compare the patients with vs. those without cancer, relevant demographic and clinical data related to cancer subtypes and time of diagnosis were retrieved from the Royal Free Hospital integrated patient records, and further information was obtained from our large SSc research database, as well as, if appropriate, from referring physicians or primary practitioners. Our database comprises information on patients with a median follow-up of 12.8 years (mean = 13.7 years; range = 0 to 52.5 years). All SSc patients are seen in our research centre at least annually. The dates of the most recent visit were recorded for all patients and were used for censoring in the analysis of time to development of cancer.

SSc onset was defined by skin sclerosis or first SSc-related internal organ manifestations other than Raynaud’s phenomenon (RP). Patient demographics and key SSc characteristics, including disease duration from the onset of both first non-RP and first RP symptoms in addition to modified Rodnan skin score were collected at baseline. Clinical details pertaining to major internal organ involvement for lung fibrosis, pulmonary arterial hypertension, renal crisis and significant gastrointestinal (GI) involvement were evaluated using consensus definitions as outlined in previously published studies [14].

Serum was routinely analysed in all SSc patients by indirect immunofluorescence, counterimmunoelectrophoresis and enzyme-linked immunosorbent assay to identify SSc-specific autoantibodies (ACAs, anti-RNAP and anti-Scl-70) at the first visit, and these analyses were repeated annually during the clinic visit. This routine ensures complete data capture for antibody reactivity and each patient has the same antibody reactivity during the disease course in our cohort [15,16]. This is consistent with published studies that indicated that these antibodies are usually mutually exclusive. Although the presence of these antibodies was not studied prior to the onset of SSc in our cohort, their specificities remained constant throughout the disease course [17]. The Hep2 immunofluorescence pattern was assessed by an experienced clinical scientist to inform further characterization of antibodies, including anti-RNAP antibodies, as described previously [17,18].

The UK NHS National Care Records Service was used to verify the vital status of patients with cancer who were otherwise lost to follow-up. Statistical methods for contingency tables (χ^2^ test or Fisher’s exact test) and time-to-event data were employed. For proportions, 95% confidence intervals (95% CI) were calculated according to Wilson’s method. Differences between SSc patients with vs. without a history of malignancy were assessed by means of Kaplan-Meier curves and the logrank test. Multiple regression analysis (logistic and Cox) was undertaken to assess the impact of autoantibody status on cancer risk (adjusted for age and sex distribution). To include the development of cancers prior to SSc onset in the analysis, these event times were rescaled by subtraction of the smallest observed event time (that is, -22 years). This step does not affect the characteristics of semiparametric Cox regression or the logrank test. For logistic regression, only the development of cancers within 36 months of SSc onset (that is, before and after onset), 36 to 60 months around SSc onset and 60 to 120 months around SSc onset were considered. Patients for whom there was insufficient follow-up were excluded, and event times beyond the preset limits were censored. Similar logistic regression analysis was undertaken for breast cancers. Hazard ratios (HRs), odds ratios (ORs) and the corresponding 95% CIs were calculated. In order to guard against type I error inflation due to multiple testing, only *P*-values below 0.01 were considered statistically significant [19]. For the purpose of data analysis, we defined the onset of SSc as the date of appearance of the first non-RP symptoms. Further analysis was undertaken using the onset of RP as a proxy for the date of SSc onset. Statistical analysis was performed using the following software: SPSS (IBM, Armonk, NY, USA), Stata (StataCorp, College Station, TX, USA; command ci) and Excel (Microsoft, Redmond, WA, USA).

## Results

### Demographic and clinical characteristics of systemic sclerosis patients with or without cancer

Among 2,177 patients with SSc who attended our centre for management of their disease, 7.1% (*n* = 154) had cancer (Table 1). Within this cohort, 85.1% (131 of 154) were females and 63.6% had lcSSc. Within this cohort, 26% had ACAs, 18.2% were positive for anti-Scl-70 antibodies and 26.6% were positive for anti-RNAP antibodies. SSc patients with cancer (median age (±SD) = 53 ± 11.9 years) were significantly older at the onset of SSc than those without cancer (45 ± 14.1 years) (*P* = 0.023). Subanalysis within antibody groups demonstrated that the age difference remained significant for patients with anti-RNAP antibodies (56 ± 9.0 years vs. 45 ± 13.7 years, respectively; *P* < 0.0001). SSc patients who had developed cancer had a significantly higher frequency of anti-RNAP antibodies than those without cancer (26.6% vs. 12.2%, respectively; *P* < 0.0001). The remaining patients with cancer harboured other antibody specificities, including antibodies against nuclear ribonucleoproteins (nRNPs) (6.5%), Pm-Scl (5.8%), Ro (5.8%) and Jo1 (0.65%). Among the SSc patients with cancer, 5.8% were negative for antinuclear antibodies (ANAs), and data were not available for five patients (two patients each with breast and lung cancer and one patient in the group of other cancers). Cancer developed after the diagnosis of SSc in 54.5% of patients (84 of 154), and 30.5% (47 of 154) had been diagnosed with cancer before visiting our centre with the diagnosis of SSc.

**Table 1:** **Demographics and clinical features of overall cohort for patients with vs. without cancer**^**a**^

**Characteristics**		**Cancer subgroups**							
	**No cancer (*n*** **= 2,023)**	**Total (*N*** **= 154)**	**Breast (*n*** **= 65)**	**Lung (*n*** **= 16)**	**Haemato (*n*** **= 19)**	**GI (*n*** **= 17)**	**GU (*n*** **= 6)**	**Gynae (*n*** **= 17)**	**Skin (*n*** **= 6)**
Sex									
Female, *n* (%)	1,663 (82.2)	131 (85.1)	65 (100)	12 (75)	14 (73.7)	13 (76.5)	2 (33.3)	17 (100)	3 (50)
Male, *n* (%)	360 (17.8)	23 (14.9)	NA	4 (25)	5 (26.3)	4 (23.5)	4 (66.7)	NA	3 (50)
SSc/cancer onset									
Median age (±SD) at SSc onset, years	45 (±14.1)	53 (±11.9)	53 (±11.1)	47 (±19.5)	51.5 (±9.4)	53 (±11.4)	59 (±18.4)	53 (±23.7)	59.5 (±19.1)
	IQR = 35 to 55	IQR = 43 to 58	IQR = 44 to 58	IQR = 41 to 54	IQR = 44 to 56	IQR = 45 to 60	IQR39 to 68	IQR = 35 to 57	IQR = 46 to 63
Cancer prior to SSc onset (median ± SD), months	No cancer	39.5 (±81.6)	36 (±77.3)	NA	109 (±88.2)	8.5 (±27.7)	25 (±74.4)	141 (±104.8)	NA
		IQR = 12 to 125	IQR = 11 to 114		IQR = 29 to 190	IQR = 1 to 48	IQR = 4	IQR = 25 to 243	
Cancer after SSc onset, (median ± SD), months	No cancer	119 (±95.3)	108 (±108.5)	125 (±93.8)	101 (±73.4)	168 (±90.6)	116.5 (±79.9)	137 (±91.9)	62 (±55.5)
		IQR = 41 to 185	IQR = 25 to 209	IQR = 71 to 200	IQR = 20 to 161	IQR = 119 to 233	IQR = 60	IQR = 20 to 194	IQR = 23 to 125
SSc subsets									
dcSSc, *n* (%)	657 (32.5)	53 (34.4)	22 (33.8)	5 (31.3)	8 (42.1)	4 (23.5)	2 (33.3)	4 (23.5)	3 (50)
lcSSc, *n* (%)	1,260 (62.3)	98 (63.6)	42 (64.6)	11 (68.8)	11 (57.9)	12 (70.6)	3 (50)	13 (76.5)	3 (50)
Missing cases, *n* (%)	106 (5.2)	3 (1.9)	1 (1.5)	0 (0)	0 (0)	1 (5.9)	1 (16.7)	0 (0)	0 (0)
SSc autoantibody status									
ACA, *n* (%)	550 (27.2)	40 (26)	17 (26.2)	2 (12.5)	4 (21.1)	8 (47.1)	1 (16.7)	6 (35.3)	1 (16.7)
Scl to 70, *n* (%)	416 (20.6)	28 (18.2)	10 (15.4)	5 (31.3)	5 (26.3)	1 (5.9)	1 (16.7)	4 (23.5)	1 (16.7)
RNAP, *n* (%)	247 (12.2)	41 (26.6)	19 (29.2)	4 (25)	5 (26.3)	3 (17.6)	2 (33.3)	3 (17.6)	3 (50)
Others, *n* (%)	515 (25.4)	31 (20)	13 (19.9)	3 (18.9)	4 (21)	3 (17.6)	1 (16.7)	4 (23.6)	1 (16.7)
ANA-negative, *n* (%)	75 (3.7)	9 (5.8)	4 (6.2)	0 (0)	1 (5.3)	2 (11.8)	1 (16.7)	0 (0)	0 (0)
Missing cases, *n* (%)	220 (10.9)	5 (3.2)	2 (3.1)	2 (12.5)	0 (0)	0 (0)	0 (0)	0 (0)	0 (0)
Organ involvement^b^									
Lung fibrosis, *n* (%)	NA	52 (33.8)	24 (36.9)	8 (50)	6 (31.6)	4 (23.5)	3 (33.3)	2 (11.8)	2 (33.3)
PAH, *n* (%)	NA	27 (17.5)	15 (23.1)	1 (6.3)	3 (15.8)	3 (17.6)	0 (0)	2 (11.8)	1 (16.7)
GI, *n* (%)	NA	85 (55.2)	35 (53.8)	9 (56.3)	12 (63.2)	9 (52.9)	1 (16.7)	10 (58.8)	3 (50)
SRC, *n* (%)	NA	16 (10.4)	4 (6.2)	1 (6.3)	2 (10.5)	4 (23.5)	1 (16.7)	2 (11.8)	0 (0)

^a^ACA, Anticentromere antibody; ANA, Antinuclear antibody; dcSSc, Diffuse cutaneous systemic sclerosis; GI, Gastrointestinal; GU, Genitourinary; Gynae, Gynaecological; Haemato, Haematological; IQR, Interquartile range; lcSSc, Limited cutaneous systemic sclerosis; NA, Not applicable; PAH, Pulmonary arterial hypertension; RNAP, RNA polymerase III; SRC, Scleroderma renal crisis; SSc, Systemic sclerosis. Cohort sample populations comprised 154 SSc patients with cancer and 2,023 SSc patients without cancer. ^b^Extent and pattern of organ involvement is defined in the Methods section.

### Associations between autoantibodies and cancer

**Figure 1:**
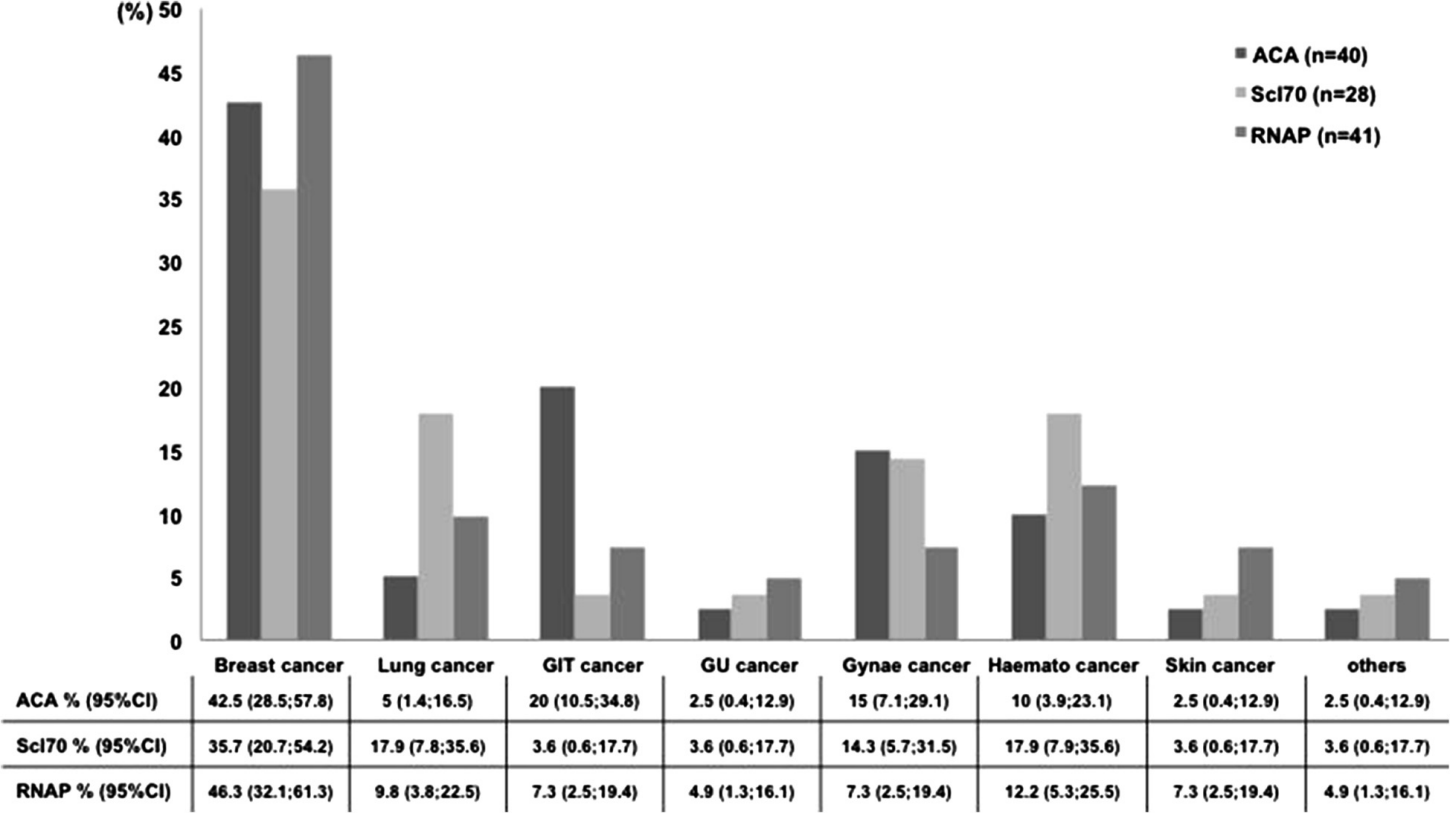
**Graph showing the frequency of different cancer subgroups for each major systemic sclerosis-specific autoantibody subtype (anticentromere, anti-Scl-70 and anti-RNA polymerase III antibodies).** ACA, Anticentromere antibody; GIT, Gastrointestinal; GU, Genitourinary; Gynae, Gynaecological; Haemato, Haematological; RNAP, RNA polymerase III. Other cancers include thyroid and brain.

The major malignancy subtypes in our cohort included breast cancer in 42.2%, lung cancers in 10.4%, haematological cancers in 12.3% and GI or gynaecological in 11%. For a number of malignancies, there was a trend towards increased frequency among patients with lcSSc. Detailed analysis of cancer subtypes was undertaken in relation to the three major SSc-specific autoantibody subgroups: ACA (*n* = 40), anti-Scl-70 (*n* = 28) and anti-RNAP (*n* = 41) (Figure 1). In our cohort, breast cancer was most frequently detected among patients with anti-RNAP antibodies (46.3% (19 of 41)), followed by patients with ACA antibodies (42.5% (17 of 40)) and anti-Scl-70 antibodies (35.7% (10 of 28)). The frequency of lung and haematological cancers (17.9%) was equally distributed in patients with anti-Scl-70 antibody. GI cancers occurred in 20% of patients with ACA. There were significantly more cancers among patients with anti-RNAP antibodies (14.2% (41 of 288)) than in patients with anti-Scl-70 antibodies (6.3% (28 of 444)) and ACA (6.8% (40 of 590)) (*P* < 0.0001 and *P* < 0.001, respectively).

Multivariable Cox regression analysis carried out to explore the association of different autoantibodies and cancer revealed that, compared to ACA (reference group), the only antibody which significantly increased the risk for cancer in SSc patients was the anti-RNAP antibody (HR = 2.96, 95% CI = 1.85 to 4.73; *P* < 0.001). For that reason, in subsequent analyses, anti-RNAP-negative patients were grouped together. Thus we found that, after being corrected for age and sex, anti-RNAP positivity is associated with a significantly increased risk of cancer, whether diagnosed either before or after SSc onset (HR = 2.55, 95% CI = 1.75 to 3.74. *P* < 0.001) (Table 2, row 2A). Older age was statistically significantly associated with cancer risk (HR = 1.46, 95% CI = 1.28 to 1.67; *P* < 0.001). There was no association, however, between sex or SSc subsets and the development of cancer in SSc patients. Subgroup analysis revealed that this association remained significant when only cancers that occurred after the onset of SSc were included (HR = 2.10, 95% CI = 1.27 to 3.48; *P* = 0.004) (Table 2, row 2B).

**Table 2:** **Multivariable Cox and logistic regression analyses to evaluate the association between anti-RNA polymerase III antibodies and development of cancers**^**a**^

**Variable**	**Frequency ( *n *)**	**Hazard/Odds ratio**	**95% CI**	***P*-value**
2A: Multivariable Cox regression analysis				
Age (per 10 years)	1,740 valid	1.46	1.28 to 1.67	<0.001
Male vs. female	299 vs. 1,441	0.83	0.50 to 1.39	0.487
RNAP^+^ vs. RNAP^-^	268 vs. 1,472	2.55	1.75 to 3.74	<0.001
2B: Multivariable Cox regression analysis				
Age (per 10 years)	1,694 valid	1.43	1.21 to 1.70	<0.001
Male vs. female	294 vs. 1,401	0.93	0.53 to 1.80	0.931
RNAP^+^ vs. RNAP^-^	250 vs. 1,445	2.10	1.27 to 3.48	0.004
2C: Logistic regression analysis				
Age (per 10 years)	1,617 valid	1.63	1.27 to 2.09	<0.001
Male vs. female	265 vs. 1,352	0.73	0.28 to 1.90	0.514
RNAP^+^ vs. RNAP^-^	249 vs. 1,368	5.83	3.11 to 10.92	<0.001

^a^CI, Confidence interval; RNAP^+^, positivity for anti-RNA polymerase III antibodies; RNAP^-^, anticentromere antibodies, anti-Scl-70, antinuclear antibody-negative and other antibodies. These analyses include all events (row 2A) with total frequency *N* = 2,177, 437 cases dropped, 128 events, 1,612 censored; events post-systemic sclerosis onset (row 2B) with total frequency *N* = 2,177, 483 cases dropped, 83 events, 1,611 censored; and for events within 36 months of systemic sclerosis onset (row 2C) with total frequency *N* = 1,623, 6 missing cases, 42 events.

**Figure 2:**
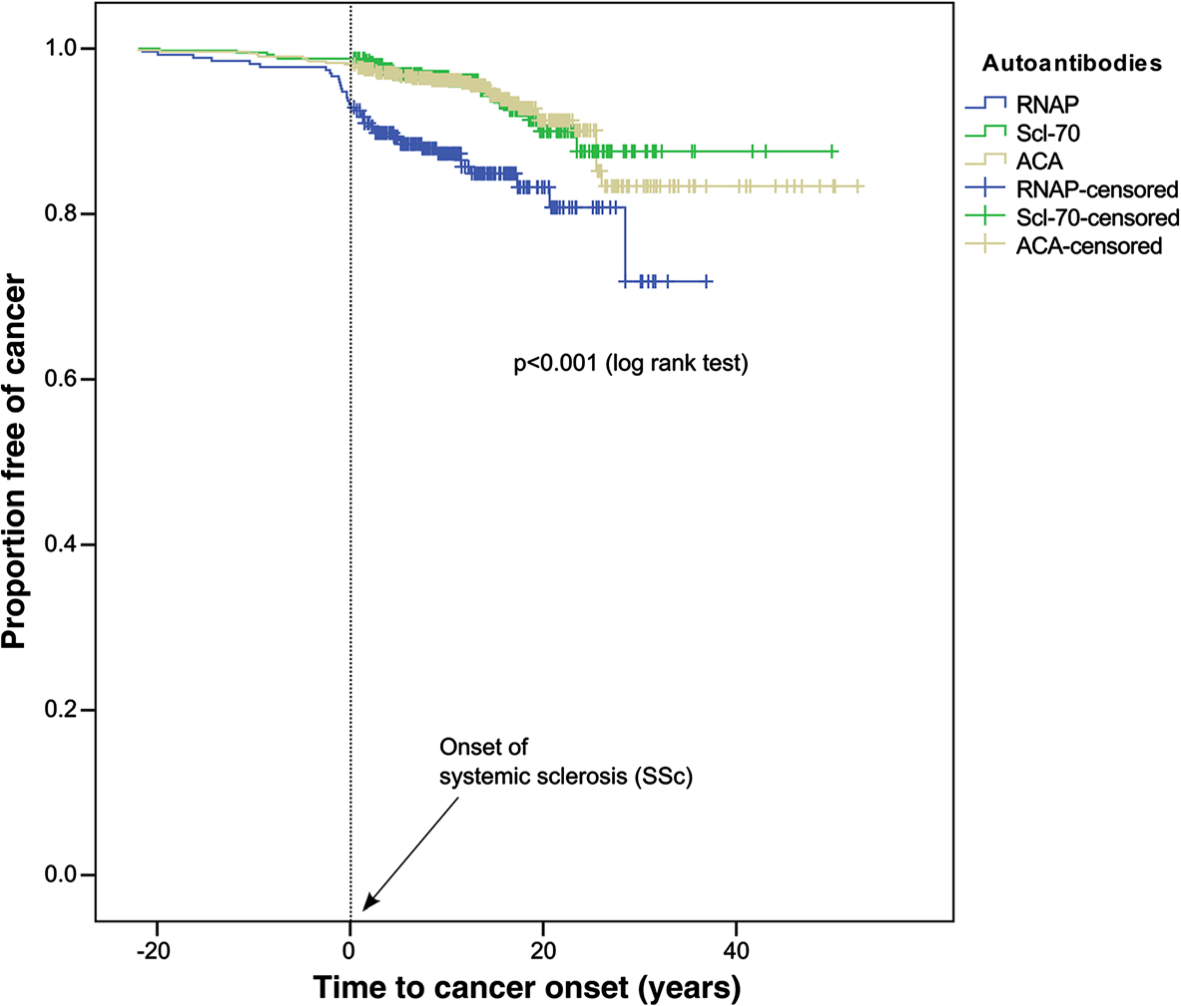
**Kaplan-Meier analysis shows three different curves for each patient group with the designated autoantibody subset.** Events (defined as diagnosis of cancer) correspond to step-downs, and censored observations (defined as most recent follow-up visit) are identified by tick marks. The plot shows a significant difference (*P* < 0.0001) between the number and timing of events between anti-RNA polymerase III (anti-RNAP)–positive patients and anticentromere antibody (ACA)– or Scl-70-positive patients. The dotted vertical line (designated as time 0) represents the clinical onset of systemic sclerosis (SSc). Within the 36-month period prior to the onset of SSc, 13 patients with the anti-RNAP antibody, compared with only 2 patients with ACA, were diagnosed with cancer. Number at risk, number and percentage loss to follow-up are represented for the intervals between SSc onset and the 20- and 40-year periods.

Kaplan-Meier estimates for the development of cancers confirmed our findings that, compared to other hallmark antibodies for SSc, anti-RNAP antibody–positive patients have a significantly higher incidence of cancers (*P* < 0.0001 by logrank test) (Figure 2). From the onset of SSc through 20 years of follow-up, 195 patients with anti-RNAP antibodies were lost to follow-up and a further 35 patients were lost to follow-up over the following 20 years. For patients with anti-Scl-70 antibody, 328 patients were lost to follow-up in the first 20 years of SSc and a further 56 patients were lost to follow-up in the following 20 years. For patients with ACA, 370 patients were lost to follow-up in the first 20 years after disease onset and another 103 patients were lost over the following 20 years. Within the cancer cohort, 103 patients were still alive (66.9%, median follow-up after cancer onset 6.4 years (25^th^ to 75^th^ percentile: 3.3 to 12.3 years), and 48 had died (*n* = 21 (13.6%) as a consequence of cancer, *n* = 4 (2.6%) due to SSc-related causes and *n* = 24 (15.6%) due to unknown causes).

### Temporal association between systemic sclerosis and cancer onset within antibody subgroups

**Figure 3:**
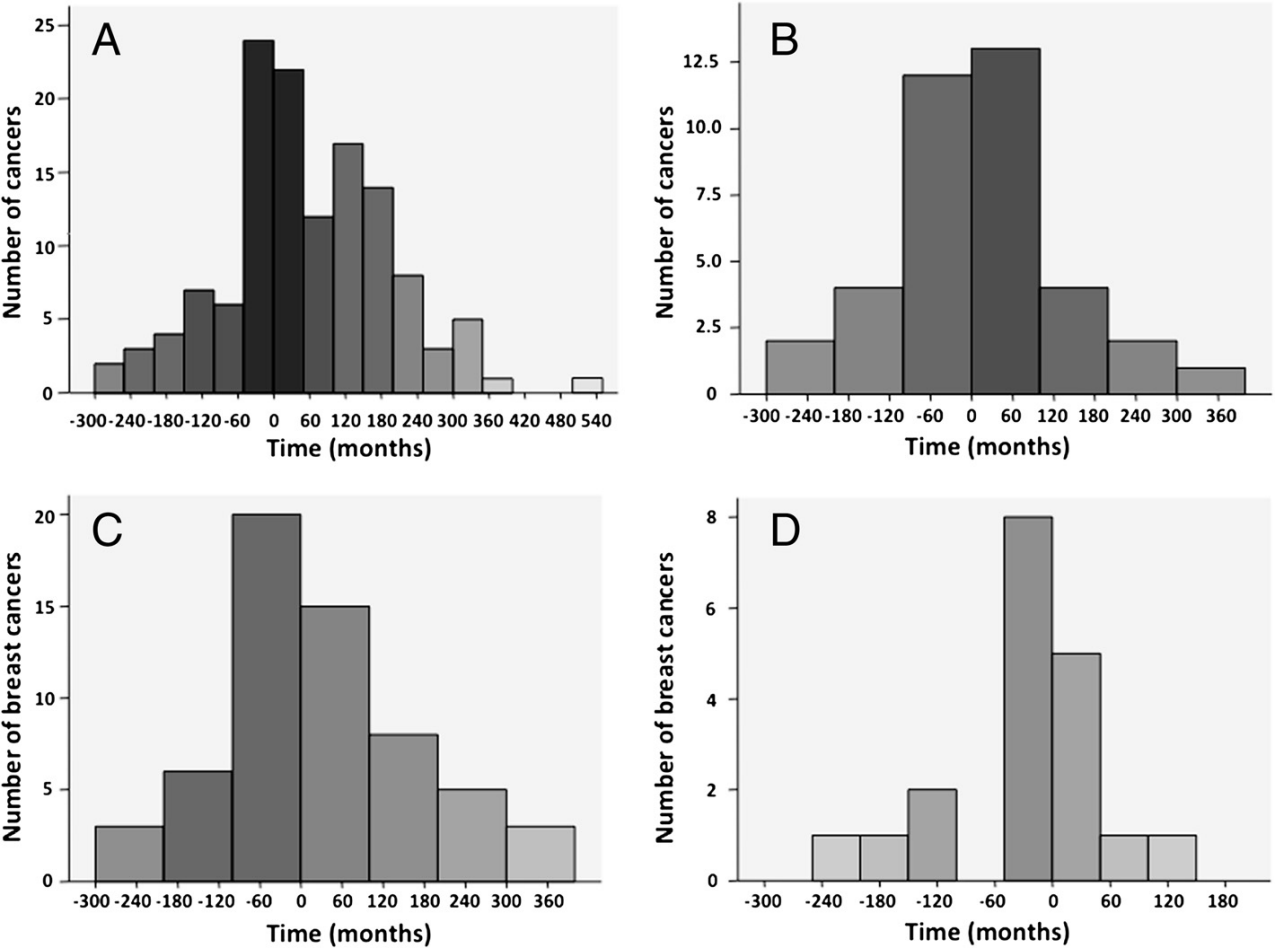
**Graphs illustrating temporal relationship of cancers (including all cancers and breast cancers) for all three major antibody reactivities and anti-RNA polymerase III antibody. (A)** Frequency of all cancers (*n* = 129) across all three major antibody reactivities (anti-Scl-70, anticentromere and anti-RNA polymerase III (RNAP) antibodies). **(B)** Frequency of all cancers for anti-RNAP antibody alone (*n* = 38). **(C)** Frequency of breast cancers (*n* = 60) for all three major antibody reactivities. **(D)** Frequency of breast cancers (*n* = 19) for anti-RNAP antibody.

Significantly more patients who harboured anti-RNAP antibodies (55.3%, 21 of 38) were diagnosed with cancer within 36 months of SSc onset compared to those with ACA (21.2%, 7 of 33; *P* < 0.004) and those with anti-Scl-70 antibodies (13.6%, 3 of 22; *P* < 0.002). Patients with anti-RNAP antibodies had nearly six times higher odds of developing cancer within 36 months compared to those without anti-RNAP antibodies (OR = 5.83, 95% CI = 3.1 to 10.9; *P* < 0.001) (Table 2, row 2C). No significant association was observed between anti-RNAP antibodies and cancer development for 36 to 60 months prior to and after SSc onset (*P* = 0.65) and between 60 and 120 months prior to and after SSc onset (*P* = 0.02). The temporal relationship of cancers for all three major antibody reactivities and anti-RNAP antibody are illustrated in Figure 3A. Most cancers occurred after the onset of SSc. The frequency was highest at the onset of SSc, and this was particularly prominent among patients with anti-RNAP antibody (Figure 3B).

### Temporal association between systemic sclerosis and breast cancer

Patients with anti-RNAP antibodies were 19 times more likely to develop breast cancer within 36 months compared to those with ACA antibodies (95% CI = 4.34 to 91.94; *P* < 0.001). No significant association was observed between anti-RNAP antibodies and the development of breast cancers 36 to 60 months prior to and after SSc onset (*P* = 0.996) and 60 to 120 months prior and after SSc onset (*P* = 0.07). Similar to the pattern observed for all cancers, the increased frequency of breast cancer appeared to cluster around the onset of SSc (Figure 3C), with its highest peak being reached just before the onset of SSc for those with anti-RNAP antibodies (Figure 3D). This pattern of cancer distribution was not observed in association with ACA or anti-Scl-70 antibodies (data not shown). In addition, age had an impact on the risk for breast cancer. The risk was doubled for each decade of life (OR = 2.14, 95% CI = 1.35 to 3.40; *P* < 0.001). No significant association was observed for the other two antibodies.

### Analysis using onset of Raynaud’s phenomenon as onset of systemic sclerosis

The duration of RP prior to progression to SSc was considered as proxy for SSc onset and included in the Cox regression analysis. Significantly more patients who harboured anti-RNAP antibodies (43.8%, 14 of 32) were diagnosed with cancer within 36 months of RP onset compared to those with ACA (13.3%, 4 of 30; *P* = 0.012) and those with anti-Scl-70 antibodies (5.3%, 1 of 19; *P* = 0.004). In addition, Cox proportional hazards regression analysis with RP onset used as a proxy for SSc onset demonstrated that the HR for development of cancer remained similar (HR = 2.86, 95% CI = 1.54 to 5.32; *P* = 0.001) in those with anti-RNAP antibodies compared to those with the ACA subtype.

## Discussion

There is emerging evidence from recent case series and epidemiological studies that SSc is associated with an increased risk for various malignancies [1,20]. In this large retrospective registry-based cohort study, we have shown that, compared with patients without cancer (*N* = 2,023), SSc patients with cancer (*N* = 154) were more frequently positive for anti-RNAP antibodies than the other hallmark SSc-specific antibodies, ACA or anti-Scl-70 antibodies. This study confirms that positivity for anti-RNAP antibodies is associated with at least a twofold increased HR for cancers that occurred before or after onset of SSc compared to those without anti-RNAP antibodies. The association remained significant for anti-RNAP antibodies and the development of cancers that occurred after the onset of SSc. In contrast, no significant differences in cancer frequency were demonstrated across disease subsets or between the sexes. Ethnicity data were not available, so this factor could not be considered in the analyses.

Importantly, the results of our present study demonstrate that positivity for anti-RNAP antibodies conferred sixfold higher odds for developing cancer within 36 months of SSc onset compared to those without this antibody, highlighting the close temporal association in this subgroup. This result supports the observation in an earlier study by Shah *et al*., who found that patients with anti-RNAP antibodies developed SSc within 2 years of cancer onset in a smaller cohort of 23 patients [8]. Airò *et al*. also reported a clustering of cancers with SSc onset in a small sample of patients with anti-RNAP antibodies [7]. In our present study, this temporal relationship was noted in particular among SSc patients with breast cancer, supporting the observation recently reported by Launay *et al*. [21]. The temporal relationship between cancer and SSc reported herein is similar to that of adenocarcinomas in dermatomyositis patients, and this association also extends, albeit with lower risk, to patients with two other autoimmune rheumatic diseases: polymyositis and systemic lupus erythematosus [22-24]. In this regard, this association suggests that the presentation of malignancy and the onset of SSc may be mechanistically linked and that the confluence of SSc and cancer also indicates that some cases of SSc might represent a paraneoplastic syndrome in which the immune system is responding to cancer.

Shah *et al*. also detected enhanced expression of nucleolar RNAP exclusively in the tumoural tissues of SSc patients with anti-RNAP antibodies in whom there was a close temporal relationship between the onset of cancer and SSc [8]. RNAP is critical for regulation of sustained cellular protein synthesis and is therefore a fundamental determinant of normal cellular growth. Recently, strong evidence has implicated abnormal RNAP activity in cancer cells from breast and lung carcinomas [25] and in fibroblasts transformed by Simian virus 40 or other polyomavirus [26,27]. It is postulated that repression of tumour suppressors p53 and retinoblastoma [28] and/or activation of oncogene product c-Myc [29] may lead to enhanced RNAP activity in malignancy. However, the biological basis for an association between specific autoantibody subtypes against NRAP and malignancy in the context of SSc is unclear. The presence of anti-RNAP antibody may initiate an antitumour immune response that, in the appropriate setting, may cross-react against specific host tissue, resulting in target tissue damage. Proof supporting this hypothesis may provide some insight into the fundamental mechanistic aspects of pathogenesis and highlight the cancer–autoimmunity interface in this particular subset of SSc as a paraneoplastic syndrome.

It is disputed whether SSc onset should be defined from the development of RP or from the first non-RP symptoms [30]. For that reason, the duration of RP prior to SSc onset was considered, and repeat analysis indicated that the association between anti-RNAP antibodies and malignancy remained significant. This finding in our study is not surprising, given that we have previously shown in an independent cohort that the duration of antecedent RP differs substantially between SSc-specific antibodies with the shortest interval between onset of RP and first non-RP features for patients with anti-RNAP antibodies (average of 1.7 years) and longest for ACA-positive patients (average of 10.8 years) [31].

Different types of cancers have been reported among SSc patients, including cancers that are common in the general population, such as breast and lung cancers. In agreement with this, the leading cancer subtypes in our patient cohort were breast cancer (42.2%), followed by haematological malignancies (12.3%), GI and gynaecological cancers (11% each), and lung cancer (10.4%). Other authors, however, have reported lung cancer [32-35] and GI malignancies [36,37] as the most common cancer subtypes associated with SSc. The variability in the reported incidence of malignancies and common cancer subtypes associated with SSc in these other studies might be due to differences in study design, case ascertainment, cancer prevalence in the general population studied, ethnicity and gender distribution. These factors may account for the high standardized incidence ratios reported for some cancer subtypes, including lung cancer. The magnitude of absolute risk for these individual cancers is likely to be low [32,38,39]. For example, the recent meta-analysis reported by Bonifazi *et al*. [20] included a large number of patients from a heterogeneous cohort and case–control retrospective studies, and the authors did not find an increased risk of breast cancers, although the results of some other studies have implicated a strong temporal clustering of breast cancer with SSc onset [40,41].

The association of breast cancer with SSc may be attributed to the potential role of sex hormones in disease development of SSc and breast cancer. Increased levels of prolactin and low dehydroepiandrosterone in patients with SSc [42,43] and breast cancer [44] lend further support to this association. It is also noteworthy that the heterogeneity of SSc may preclude a definitive conclusion regarding the association of SSc and cancer.

A variety of potential reasons have been postulated for the apparent association of SSc and cancer. These include shared risk factors such as gender distribution, exposure to immunosuppressive drugs and possible shared genetic backgrounds of both disorders. The precise pathogenic mechanisms that cause and maintain an autoimmune response in patients with cancer has not been fully elucidated. Autoimmunity in cancer may occur as a consequence of an abnormal self-antigen expression and/or mutated antigens, as well as of tumour-derived biological substances produced by cancer cells. In addition, the consequences of chronic inflammation and fibrotic processes may damage tissue, cells and DNA, leading to altered immune responses and the development of malignant transformation, triggering the development of lung cancer or cutaneous malignancies. Dysregulation of molecular signalling pathways involving transforming growth factor β and SMAD pathways have been reported to induce fibrosis and also tumourigenesis [45]. In a recent study, the investigators suggested that both DNA methylation and histone modification may contribute to excessive synthesis of extracellular matrix proteins in SSc and that an imbalance may lead to autoimmune diseases and also cancer [46].

## Conclusions

Our study, which to the best of our knowledge is the largest to date addressing the temporal association between the autoantibody subtypes and cancer in SSc, provides independent confirmation to recent studies that SSc patients who harbour anti-RNAP antibodies have an increased risk of cancer within a short interval of the clinical onset of SSc. Increased awareness among physicians is required to institute appropriate investigations where necessary. Further studies to evaluate the biological link between anti-RNAP antibodies and malignancy in SSc should be undertaken in future studies. Our data add to the early observation, that SSc may be a paraneoplastic syndrome in some cases. Currently, there are no data to support the cost-effectiveness of screening for underlying malignancy in patients with early onset SSc with anti-RNAP antibodies. However, it may be appropriate to screen individuals at high risk and those who fail to respond to therapy.

## Abbreviations

ACA: Anticentromere antibody; ANA: Antinuclear antibody; CI: Confidence interval; dcSSc: Diffuse cutaneous systemic sclerosis; GI: Gastrointestinal; GU: Genitourinary; Gynae: Gynaecological; Haemato: Haematological; HR: Hazard ratio; lcSSc: Limited cutaneous systemic sclerosis; RNAP: RNA polymerase III; RP: Raynaud’s phenomenon; SSc: Systemic sclerosis.

## Competing interests

The authors declare that they have no competing interests.

## Authors’ contributions

PM, VO and CD conceived and designed the study. PM, CF, CC, CD, AS and VO were involved in data acquisition. MH made particular contributions to the statistical analyses and interpretation of data. All authors contributed to the analysis and interpretation of the data and jointly wrote and approved the final manuscript.
